# Oligometastatic deposits of prostate cancer found within the sigmoid pericolic fat that was resected for colonic adenocarcinoma: a case report

**DOI:** 10.1186/s13256-022-03441-4

**Published:** 2022-06-05

**Authors:** David N. Naumann, Rahul K. Hejmadi, Jonathan J. R. Richardson

**Affiliations:** 1 Department of Surgery, University Hospitals Birmingham NHS Foundation Trust, Queen Elizabeth Hospital Birmingham, Mindelsohn Way, Edgbaston, Birmingham, B15 2TH UK; 2 Department of Cellular Pathology, University Hospitals Birmingham NHS Foundation Trust, Queen Elizabeth Hospital Birmingham, Mindelsohn Way, Edgbaston, Birmingham, B15 2GW UK

**Keywords:** Colorectal cancer, Metastases, Prostate cancer, Colorectal resection, Lymph nodes

## Abstract

**Background:**

Prostate cancer may rarely metastasize to the colon and colonic lymph nodes, and local treatment of oligometastatic deposits may improve oncological outcomes. Immunohistochemical stains are used to determine the most likely source of metastatic deposits when they are seen within surgical specimens. The aim of this case report is to illustrate how such techniques were used to identify unexpected prostatic metastases within the pericolic fat of a sigmoid colon resection specimen following elective curative surgery for colorectal cancer. To our knowledge, this is the first report of complete excision of oligometastatic deposits of prostate cancer found incidentally within the specimen of another cancer.

**Case report:**

An 89-year-old Caucasian man underwent sigmoid colectomy for an obstructing colorectal cancer in the sigmoid colon with some mesenteric lymphadenopathy. He had previously received radical radiotherapy for prostate cancer 10 years earlier. When the specimen was examined by the histopathologist, it was noted that the pericolic fat adjacent to the colorectal adenocarcinoma contained some metastatic deposits. Positive immunohistochemical staining for prostate-specific antigen and prostate-specific acid phosphatase with negative staining for CDX2 and CK20 revealed these to be prostatic metastases rather than colonic. Since these were completely excised, and there were no other metastases, this represented a serendipitous, curative excision of oligometastatic deposits of an additional cancer to the one that was being treated.

**Conclusions:**

This case illustrates how immunohistochemical staining may be used to distinguish the source of metastatic deposits based on the likelihood of primary tumor from a careful and thorough patient history.

## Background

Immunohistochemical staining techniques are used to determine the most likely source of metastatic deposits when they are seen within surgical specimens. The choice of stains is made based on the most likely candidates for the primary malignancy. When unexpected metastases are discovered, a careful and detailed past medical history is essential to aid in the selection of appropriate, targeted tests. The aim of this case report is to illustrate how such techniques were used to identify unexpected oligometastatic prostate cancer within a surgical specimen following elective curative surgery for colorectal cancer.

## Case presentation

An 89-year-old Caucasian man who lived independently with good exercise tolerance was referred to colorectal services with a computed tomography (CT) finding of an obstructing mass in the sigmoid colon with some mesenteric lymphadenopathy. There were features consistent with colorectal cancer on flexible sigmoidoscopy. There were no metastases seen on a staging CT of his chest, abdomen, and pelvis. No positron emission tomography-CT was undertaken. He had radical radiotherapy for prostate cancer 10 years earlier, with a recent biochemical relapse that was being managed with hormonal therapy. His latest prostate-specific antigen (PSA) was 1.8 ng/ml. After fully discussing all options for management of his sigmoid tumor, the patient opted for management with a colonic stent. However, this did not relieve his symptoms and the stent became occluded. He therefore underwent a sigmoid colectomy with end colostomy. The inferior mesenteric artery was ligated, and a colonic specimen from descending colon to the upper rectum was resected along with the corresponding mesocolon. There were no peritoneal deposits, and no evidence of metastatic disease intraoperatively. He was discharged on the fourth postoperative day without complications. The specimen was sent for histological examination.

**Fig. 1:**
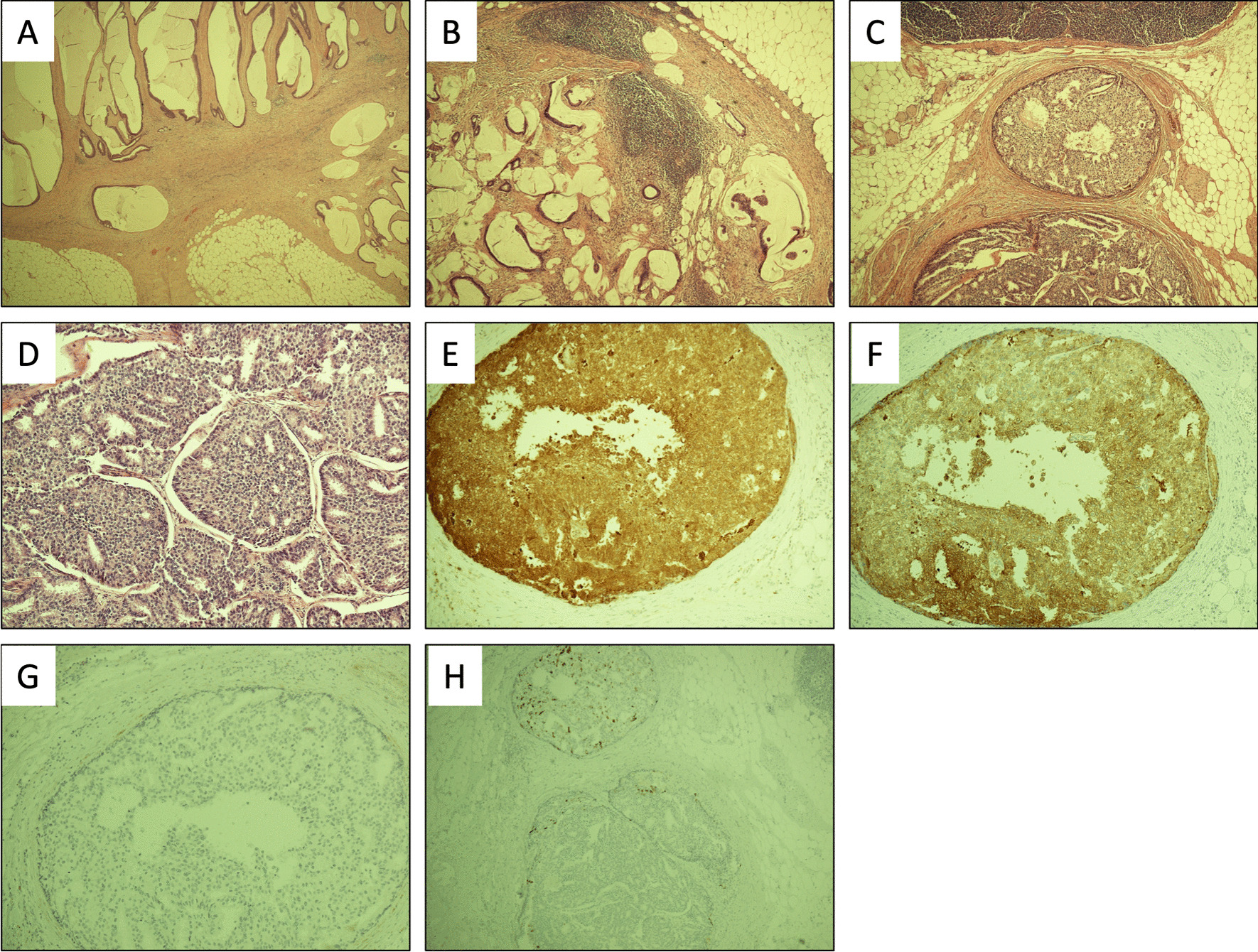
Histopathology from the colonic specimen depicting **A** mucinous colonic adenocarcinoma in mesenteric fat, **B** nodal metastases (mucinous colonic adenocarcinoma), **C**, **D** deposits of metastatic prostate adenocarcinoma, **E** PSA immunohistochemistry positive confirming prostate adenocarcinoma metastases, **F** PSAP immunohistochemistry positive confirming prostate adenocarcinoma metastases, **G** CDX2 immunohistochemistry negative, excluding gastrointestinal tract metastases, and **H** CK20 immunohistochemistry, very focally positive, excluding a gastrointestinal tract primary

When the specimen was examined, there was mucinous colonic adenocarcinoma in the mesenteric fat (Fig. 1a) with some further nodal metastases of the same in 1 out of 27 sampled lymph nodes (Fig. 1b). There were also separate, well-defined metastatic tumor deposits comprising smaller, cuboidal cells embedded within the pericolic fat of the mesocolon that differed from the tall columnar cells seen in colorectal cancer (Fig. 1c, d). Due to the patient’s previous history of prostate cancer, immunohistochemical staining for PSA and prostate-specific acid phosphatase (PSAP) was undertaken and was positive (Fig. 1e and f respectively). Furthermore, CDX2 and CK20 immunochemistry confirmed that these cells were not from the gastrointestinal tract (Fig. 1g and h, respectively). The colorectal margins were clear, and the final histological staging was T_4_ N_1_ M_0_ V_1_ R_0_, with the presence of incidental oligometastatic deposits of prostate cancer within the pericolonic fat. There was no evidence of any other disseminated prostatic metastases.

## Discussion and conclusions

Prostate cancer most commonly metastasizes to bone, but may also metastasize to distant lymph nodes, liver, thorax, brain, kidneys and adrenals, the retroperitoneum, and digestive tract [1]. There are some reports in literature of colonic masses mimicking colorectal cancer and polyps that turned out to be prostatic metastases [2–7]. There have also been some reports of prostatic metastases to colorectal lymph nodes [8, 9]. However, to our knowledge, our patient is the first in the literature to have had a focus of prostate cancer metastasis found incidentally within the pericolic fat of a colorectal cancer specimen without involvement of the colonic or lymph node tissue. Since these were solitary metastatic deposits without further dissemination, this may have represented a successful resection of previously undetected oligometastatic disease entirely by chance. There is some evidence of improved patient outcomes after resection of oligometastatic prostate cancer [10], but this is controversial [11] and it is unknown whether our patient will benefit from this serendipitous event.

Patients who have had previous radiotherapy for prostate cancer are at higher risk of developing colonic or rectal cancers [12]. It is not possible to know whether our patient’s colorectal cancer was a consequence of radiotherapy a decade earlier, but the coincidental prostatic metastases adjacent to colorectal adenocarcinoma is intriguing. Immunohistochemical staining was selected according to the likelihood of prostatic disease based on the previous history of the patient. This illustrates the importance of a thorough knowledge of the past medical history of patients undergoing surgical resection within the framework of a multidisciplinary team discussion. 

We report the presence of coincidental metastatic prostate cancer within the pericolic fat of a colorectal cancer resection in a patient who had radical radiotherapy for prostate cancer 10 years earlier. This case demonstrates the versatility of the biology of prostatic cancer, and the importance of having a detailed patient history, to select appropriate immunohistochemical staining for unexpected findings within surgical specimens.

## Data Availability

Further information can be obtained from the authors on reasonable request.

## Abbreviations

CT: Computed tomography
PSA: Prostate-specific antigen
PSAP: Prostate-specific acid phosphatase
